# Differential pulmonary effects of CoO and La_2_O_3_ metal oxide nanoparticle responses during aerosolized inhalation in mice

**DOI:** 10.1186/s12989-016-0155-3

**Published:** 2016-08-15

**Authors:** Jennifer D. Sisler, Ruibin Li, Walter McKinney, Robert R. Mercer, Zhaoxia Ji, Tian Xia, Xiang Wang, Justine Shaffer, Marlene Orandle, Amy L. Mihalchik, Lori Battelli, Bean T. Chen, Michael Wolfarth, Michael E. Andrew, Diane Schwegler-Berry, Dale W. Porter, Vincent Castranova, Andre Nel, Yong Qian

**Affiliations:** 1 Health Effects Laboratory Division, National Institute for Occupational Safety and Health, 1095 Willowdale Road, Morgantown, WV 26505 USA; 2 Department of Pharmaceutical Sciences, West Virginia University, Morgantown, WV 26506 USA; 3 Division of NanoMedicine, Department of Medicine, University of California, 10833 Le Conte Ave, Los Angeles, CA 90095 USA; 4 Center for Environmental Implications of Nanotechnology, University of California, Los Angeles, CA 90095 USA; 5 California NanoSystems Institute, University of California, 570 Westwood Plaza, Los Angeles, CA 90095 USA; 6 School for Radiological and Interdisciplinary Sciences (RAD-X), Collaborative Innovation Center of Radiation Medicine of Jiangsu Higher Education Institutions, Soochow University, Suzhou, 215123 China

**Keywords:** Nanoparticles, Metal oxides, Pulmonary response, In vivo, Mouse

## Abstract

**Background:**

Although classified as metal oxides, cobalt monoxide (CoO) and lanthanum oxide (La_2_O_3_) nanoparticles, as representative transition and rare earth oxides, exhibit distinct material properties that may result in different hazardous potential in the lung. The current study was undertaken to compare the pulmonary effects of aerosolized whole body inhalation of these nanoparticles in mice.

**Results:**

Mice were exposed to filtered air (control) and 10 or 30 mg/m^3^ of each particle type for 4 days and then examined at 1 h, 1, 7 and 56 days post-exposure. The whole lung burden 1 h after the 4 day inhalation of CoO nanoparticles was 25 % of that for La_2_O_3_ nanoparticles. At 56 days post exposure, < 1 % of CoO nanoparticles remained in the lungs; however, 22–50 % of the La_2_O_3_ nanoparticles lung burden 1 h post exposure was retained at 56 days post exposure for low and high exposures. Significant accumulation of La_2_O_3_ nanoparticles in the tracheobronchial lymph nodes was noted at 56 days post exposure. When exposed to phagolysosomal simulated fluid, La nanoparticles formed urchin-shaped LaPO_4_ structures, suggesting that retention of this rare earth oxide nanoparticle may be due to complexation of cellular phosphates within lysosomes. CoO nanoparticles caused greater lactate dehydrogenase release in the bronchoalveolar fluid (BALF) compared to La_2_O_3_ nanoparticles at 1 day post exposure, while BAL cell differentials indicate that La_2_O_3_ nanoparticles generated more inflammatory cell infiltration at all doses and exposure points. Histopathological analysis showed acute inflammatory changes at 1 day after inhalation of either CoO or La_2_O_3_ nanoparticles. Only the 30 mg/m^3^ La_2_O_3_ nanoparticles exposure caused chronic inflammatory changes and minimal fibrosis at day 56 post exposure. This is in agreement with activation of the NRLP3 inflammasome after in vitro exposure of differentiated THP-1 macrophages to La_2_O_3_ but not after CoO nanoparticles exposure.

**Conclusion:**

Taken together, the inhalation studies confirmed the trend of our previous sub-acute aspiration study, which reported that CoO nanoparticles induced more acute pulmonary toxicity, while La_2_O_3_ nanoparticles caused chronic inflammatory changes and minimal fibrosis.

**Electronic supplementary material:**

The online version of this article (doi:10.1186/s12989-016-0155-3) contains supplementary material, which is available to authorized users.

## Background

Nanotechnology is perceived as one of the most notable research achievements of this century. Engineered nanoparticles are currently being used in many industrial areas. Particularly, metal oxide nanoparticles are an important class of engineered nanomaterials with broad applications in many industries. From a health perspective, previous studies have shown that some metal oxide nanoparticles can induce adverse effects at organ, tissue, cellular, protein, and gene levels [1]. A number of transition metal oxide nanoparticles exhibit semi-conductor properties in which the material band gap plays a role in electron transfer to and from biological redox components. This can lead to the generation of adverse health effects due to the generation of oxygen radicals and oxidative stress [2]. According to the band gap theory, electron transfer between metal oxide nanoparticles and aqueous biological reactants is dependent on an overlap of the metal oxide band gap energy level with a biological equivalent, known as the cellular redox potential [3]. The accompanying electron transfer from the nanoparticle surface to a series of redox couples that reconstitute of biological redox potential can result in the generation of reactive oxygen species (ROS), disturbance of cellular redox homeostasis, and oxidative damage to DNA or proteins [4, 5]. Cobalt monoxide (CoO) and lanthanum oxide (La_2_O_3_) differ with respect to their biological properties. While for the transition metal oxide, CoO, the conduction band energy overlaps with the cellular biological redox potential, there is no similar overlap for the rare earth oxide, La_2_O_3_ [3]. In contrast, La_2_O_3_ (and other rare earth oxide) nanoparticles exhibit non-oxidative surface reactivity that may come into play in a biological environment with the possibility to generate hazard through complexation of cellular phosphate groups in the lysosome [6]. In brief, this study addresses how phosphate complexation of La nanoparticles in the acidic environment of the phagolysosome can induce lysosomal membrane damage, activation of the NRLP3 inflammasome, and IL-1β production [6].

Previously, we investigated CoO and La_2_O_3_ nanoparticle-induced cellular toxicity, production of superoxide radicals, and alterations in gene expression related to oxidative stress and cellular death in human small airway epithelial cells (SAEC) [7]. Results in that study showed that CoO nanoparticles caused more adverse effects than La_2_O_3_ nanoparticles, using cytotoxicity assays in SAEC. Moreover, CoO nanoparticles produced more superoxide radicals and induced greater induction of total global tyrosine and threonine phosphorylation in SAEC compared to the La_2_O_3_ nanoparticle. Profiling of oxidative stress pro-apoptotic gene expression demonstrated that CoO nanoparticles induced significant transcriptional activation of PTGS2(COX2), NOS2, SOD3, and MT3 genes in SAEC. These results indicate that different modes of action could be involved in mediating cellular toxicity by CoO and La_2_O_3_ nanoparticles. Similar observations regarding the cytotoxic potential of CoO and La_2_O_3_ nanoparticles have been reported by other laboratories. For instance, CoO nanoparticles have been shown to be cytotoxic in human lymphocytes, alveolar A549, and bronchial BEAS-2B epithelial cells [8, 9]. However, Lim et al. showed that La_2_O_3_ nanoparticles were cytotoxic at high doses and prolonged exposure times in RAW264.7 and A549 cells [10]. In addition, our recent study indicates that La_2_O_3_ nanoparticles (50 μg/mL) induced lysosomal damage, cathepsin B release, and IL-1β production after in vitro exposure of differentiated THP-1 macrophages for 6 h [6]. Taken together, these data demonstrate that CoO and La_2_O_3_ nanoparticles have the potential to induce different biological effects in vivo, based on different modes of action after pulmonary exposure. For instance, in oropharyngeal aspiration studies in mice, we observed more acute inflammation with CoO and La_2_O_3_ nanoparticles which is in agreement with in vitro cytotoxicity results [3]. However, in a longer term (21 day post exposure) La_2_O_3_ aspiration study, we observed elevation of fibrogenic factors in bronchoalveolar lavage fluid (BALF) and increased lung collagen in mice; which is in agreement with in vitro activation of the NLRP3 inflammasome in cellular studies [6].

The objective of the present study was to explore the pulmonary effects of CoO and La_2_O_3_ nanoparticles in mice for up to 56 days after whole-body inhalation exposure, which is more representative of possible human exposures than a bolus dose administration. The rationale for this study was to determine whether the different modes of action of CoO and La_2_O_3_ nanoparticles could be seen to elicit different types of information after short and longer-term exposure as a reflection of the effect of the bandgap overlap and pro-oxidative properties of the former particle type versus activation of the NRLP3 inflammasome by La_2_O_3_ nanoparticles.

BALF and pulmonary tissue were analyzed to determine if these nanomaterials have overlapping or contrasting features that can be related to inhalation exposure at low and high doses. We demonstrate that there are indeed differences in the pulmonary responses to CoO and La_2_O_3_ nanoparticles in the induction of acute vs. chronic pulmonary responses. Such differences are reflective of adverse outcome pathways (AOPs) for these materials [11], which we previously established by our cellular and bolus aspiration studies [3, 6, 7]. The results of this inhalation study support the usefulness of a tiered approach including mechanistically relevant in vitro screening tests and bolus in vivo exposures in predicting pulmonary responses during inhalation exposures to nanoparticles [11].

## Methods

### Nanoparticles

CoO nanoparticles were purchased from SkySpring Nanomaterials (Houston, TX) and La_2_O_3_ nanoparticles were purchased from Nanostructured & Amorphous Materials, Inc. (Houston, TX). The physicochemical characteristics of these particles are given in Table 1 [3, 7].

**Table 1:** Physiochemical Characterization of CoO and La_2_O_3_ Nanoparticles

Property	Characterization Technique	Nanoparticles	
		CoO	La2O3
Size	TEM	72 ± 16 nm	25 ± 5 nm
Crystal Structure	XRD	Cubic	Hexagonal
Morphology	TEM	Agglomerate	Agglomerate
Size in DI H_2_O	DLS	185 ± 12 nm	211 ± 11 nm
Zeta potential in DI H_2_O	Zeta PALS	32 ± 4 mV	58 ± 7 mV

### Acellular transformation of La_2_O_3_ nanoparticles in phagolysosomal simulated fluid (PSF)

La_2_O_3_ nanoparticles were suspended in 10 mL of phagolysosomal simulated fluid (PSF) (142 mg/L Na_2_HPO_4_, 6.65 g/L NaCl, 62 mg/L Na_2_SO_4_, 29 mg/L CaCl_2_ • 3 H_2_O, 250 mg/L glycine, 8.09 g/L potassium phthalate, pH 4.5) at a concentration of 50 μg/mL, and sonicated at 32 W for 15 s. Following incubation at 37 °C for 24 h, the particle suspensions were centrifuged at 15,000 rpm for 10 min. The pellets were collected, thoroughly washed with DI H_2_O and dried for further use. The composition and morphology of PSF-treated La_2_O_3_ nanoparticles were characterized by XRD (Panalytical X’Pert Pro diffractometer, Cu KR radiation) and TEM (JEOL 1200 EX, accelerating voltage 80 kV), respectively.

### Cathepsin B release and IL-1β production in THP-1 cells

THP-1 cells were differentiated into macrophages as described previously [6]. Briefly, aliquots of 5 × 10^4^ cells were seeded in 0.1 mL complete medium and primed with 1 μg/mL of phorbol 12-myristate acetate (PMA) overnight in 96-well plates (Corning; Corning, NY, USA). These macrophages were treated with CoO or La_2_O_3_ nanoparticles (50 μg/mL) in complete RPMI 1640 for 6 h, followed by washing in phosphate-buffered saline (PBS). Cells were stained with the cathepsin B substrate, Magic Red (ImmunoChemistry Technologies), for 1 h and fixed in 4 % paraformaldehyde for 20 min. Following washing in PBS, nuclei were stained with Hoechst 33342 for 1 h. The cells were visualized under a confocal microscope (Leica Confocal SP2 1P/FCS). High magnification images were obtained under the 63× objective and the lysosomal vs. cytosolic staining of cathepsin B noted as an indication of particle-induced lysosomal damage.

IL-1β production was detected in the THP-1 differentiated macrophage culture medium, using a human IL-1β ELISA Kit (BD; San Jose, CA, USA). Briefly, cells (5 × 10^4^ cells) were treated with 0 – 100 μg/mL CoO or La_2_O_3_ nanoparticles suspended in complete RPMI 1640 that also contained 10 ng/mL of lipopolysaccharide (LPS) to induce production of pro-IL-1β. The nanoparticle suspensions were sonicated in media containing 10 % fetal bovine serum with a Sonics & Materials probe at 32 W for 15 s before adding to the cells. As reported previous studies [3], acellular suspensions of CoO nanoparticles generated significant levels of reactive oxygen species (ROS) measured as DCF fluorescence. ROS generation was not affected by sonication (Additional file 1: Figure S1). In contrast, suspensions of La_2_O_3_ nanoparticles did not generate ROS either before or after sonication (Additional file 1: Figure S1). It should be noted that the densities of CoO and La_2_O_3_ nanoparticles and their resultant sedimentation rates in cell culture media are similar. Calculations indicate that 80 % of the nanoparticles would settle on the cells after 24 h.

### Animals

Eight week old male, pathogen-free C57BL/6 J mice were obtained from Jackson Laboratories (Bar Harbor, ME). Mice were housed individually and allowed to acclimate for 1 week in an AAALAC-accredited animal facility and 4 days within the chambers, which were used later for whole-body inhalation exposure. After exposure, animal weight was monitored weekly to assess the health of the animals. All procedures performed on animals were approved by the NIOSH Institutional Animal Care and Use Committee (IACUC).

Mice were assigned to exposure groups using a randomized complete block design. Mice were exposed to either CoO or La_2_O_3_ nanoparticles, or air control. Mice were used to determine whole lung deposition immediately post exposure, lung clearance, inflammatory and damage markers in bronchoalveolar lavage, or histopatholoic responses. Animals were exposed to a low dose (10 mg/m^3^) or high dose (30 mg/m^3^) for 4 days at 6 h/day and were sacrificed at 1 h and 1, 7, or 56 days post exposure. At 1 h post exposure, animals were taken for lung burden (*n* = 5 mice per each CoO and La_2_O_3_ exposure group and air control). At 1, 7, and 56 days post exposure, animals were analyzed for bronchoalveolar lavage and histopathology (*n* = 12 for each CoO and La_2_O_3_ exposure group and *n* = 24 for air control). At 56 days post exposure, mice were taken for analysis of nanoparticle clearance from the mouse lung (*n* = 5 mice per each CoO and La_2_O_3_ nanoparticle exposure group).

### Inhalation exposure system

The CoO and La_2_O_3_ nanoparticles aerosols were generated using an acoustical-based, computer controlled, whole body inhalation system as described previously [12]. In brief, the inhalation exposure system combines air flow controllers, aerosol particle monitors, data acquisition devices, and custom software with automated feedback control to achieve constant and repeatable exposure chamber temperature, relative humidity, pressure, aerosol concentration, and particle size distributions as previously described [12–15]. The system is capable of producing airborne particles continuously for long periods of time with minimal fluctuations.

In this study, the CoO and La_2_O_3_ nanoparticles aerosol mass concentrations within the exposure chamber were continuously monitored with a Data RAM (DR-40000 Thermo Electron Co, Franklin, MA), and gravimetric determinations (37 mm cassettes with 0.45 μm pore-size Teflon filters) were used to calibrate and verify the Data RAM readings each day. The target concentrations for both CoO and La_2_O_3_ were 10 mg/m^3^ for a duration of 6 h/day for 4 consecutive days (low dose), or 30 mg/m^3^ for a duration of 6 h/day for 4 consecutive days (high dose). These aerosol concentrations and exposure durations were chosen to yield lung burdens in the range of our previous aspiration study [6]. Each exposure group had a corresponding clean air control exposed group.

The mass-based particle size distribution of the nanoparticle aerosol in the exposure chamber was determined by a cascade impactor (MOUDI Model 110-R, MSP Corp., Shoreview, MN). Exposure chamber air (10 L/min) was mixed with filtered air (20 L/min) and fractionated onto 10 size stages of the impactor, representing 10 size ranges from 56 nm to 18 μm. Mass median aerodynamic diameter was obtained from these data.

The count-based particle size distribution of particles within the exposure chamber was measured using a Scanning Mobility Particle Sizer (SMPS model, TSI Inc., Shoreview, MN) at a sampling rate of 0.3 L/min. Count median aerodynamic diameter was obtained from these data assuming a spherical particle shape.

Aerosol samples from the exposure chamber were collected on 25 mm polycarbonate filters with 0.2 μm pores (EMD-Millipore, Billercia, MA) at a rate of 1 L/min to obtain particle samples for further analysis by field emission scanning electron microscopy (FESEM) as described previously [12, 14].

### Lung burden and lung clearance

Mice were euthanized after 1 h or 56 days post-exposure by intraperitoneal (i.p.) injection of sodium pentobarbital and exsanguination. The 1 h analysis time was included to determine lung burden immediately following the 4 day inhalation exposure. This would provide information concerning the rapid clearance of particles from the conducting zone during the 4 days of inhalation exposure. The differences in lung burden from 1 h to 56 days post exposure would provide information concerning the slow phase of clearance. Lungs with associated lymph nodes (whole lungs) were harvested from nanoparticle-exposed mice, as well as, ambient air control mice, snap frozen in liquid nitrogen, and stored at −80 °C. CoO or La_2_O_3_ nanoparticle lung burden was determined by Elemental Analysis, Inc. (Lexington, KY) using instrumental neutron activation analysis (INAA). Briefly, lung samples were exposed to a thermal neutroflux of 5 × 10^3^ n/cm^2^/s for 24 h to produce radioactive Co or La. The radioactive emission from the samples was measured by gamma-ray spectroscopy. The intensity of emissions for these lung samples was compared to spiked samples and is indicative of the amount of the Co or La present in the lung.

### Bronchoalveolar lavage

At 1, 7 and 56 days post exposure, mice were euthanized by i.p. injection of sodium pentobarbital (>100 mg/kg body weight). These post exposure times were chosen to determine if lung damage and inflammation were transient or more prolonged as determined by markers in bronchoalveolar lavage (BAL) samples as described previously [14]. BAL of the whole lung was performed by inserting a cannula into the trachea and lavaging with Ca^2+^ and Mg^2+^ free phosphate-buffered saline, pH 7.4 with the addition of 0.55 mM D-glucose (PBS). The initial whole lung lavage was performed twice using 0.6 mL PBS. The subsequent lavages were performed using a 1 mL aliquot per lavage until a total collection volume of 4 mL PBS was obtained. BAL cells were collected through centrifugation (1,500 × g, 5 min, 4 °C) of the first and subsequent lavages, cell pellets were combined, washed with 1 mL PBS, and centrifuged (1,500 × g, 5 min, 4 °C). BAL cells were re-suspended with 250 μL PBS and total cell counts were determined using a Coulter Counter (Multisizer 4, Beckman Coulter, Brea CA). Cytospins of the BAL cells were generated using a Shandon cytospin 4 (Thermo Fisher, Waltham, MA) and stained with modified Wright stain (Thermo Fisher, Waltham, MA). Cytospins were analyzed for cell differentials using an Olympus DP73 light microscope (Tokyo, Japan) and analyzed using the Olympus CellSens Dimension program (Tokyo, Japan). A total of 200 cells per slide were counted. The total number of each cell type was determined from total cell counts previously determined. The supernatant of the first lavage was collected after centrifugation and used for lactate dehydrogenase (LDH) levels and cytokine/chemokine analysis.

### BAL fluid LDH measurement

BAL fluid (BALF) was analyzed for lactate dehydrogenase (LDH) activity, a measure of cytotoxicity, using a COBAS MIRA Plus (Roche Diagnostic Systems, Montclair, NI) as previously described [14]. Samples (never frozen) were assayed via manufacturer’s protocol where the chemical oxidation of LDH to pyruvate coupled with the reduction of NAD^+^ was measured at 340 nm.

### BAL fluid cytokine and chemokine measurement

BALF from 1, 7, and 56 post exposure day mice was used to analyze cytokine and chemokine expression as previously described [14] with the following modifications. Samples were processed with the V-Plex pro-inflammatory Panel (mouse) kit (Meso Scale Discovery, Gaithersburg, MD) using the manufacturer guidelines. Samples were run in duplicate. The following cytokine/chemokines were analyzed: interferon gamma (IFN-γ), interleukin-10 (IL-10), interleukin-12 (IL-12p70), interleukin-1 beta (IL-1β), interleukin-2 (IL-2), interleukin-4 (IL-4), interleukin-5 (IL-5), interleukin-6 (IL-6), neutrophil-activating protein 3 (KC/GRO), and tumor necrosis factor-alpha (TNFα).

### Fixation and sectioning of lung tissue

At 1, 7, and 56 days post exposure, mice from the 10 and 30 mg/m^3^ groups were euthanized by an i.p. injection with sodium pentobarbital (>100 mg/kg body weight) followed by exsanguination. These time points provide histopathological information concerning the time course of particle-induced inflammation. The slow development of fibrosis would be evaluated by collagen staining of lung tissue at 56 days post exposure. The left lung was inflated with 0.5 mL 10 % neutral buffered formalin. Tracheobronchial lymph nodes were taken from 1 and 56 day post exposure mice from the air control and 30 mg/m^3^ CoO or La_2_O_3_ nanoparticle groups and fixed with 10 % neutral buffered formalin. Sections of the left lung and the tracheobronchial lymph nodes were processed, embedded in paraffin, cut at 5 microns and stained with hematoxylin and eosin (H&E) for microscopic analysis. Sections of the left lung were also stained with Masson’s trichrome and Sirius red for fibrosis evaluation. Stained slides were sent to Integrated Laboratory Systems, Inc. (Research Park Triangle, NC) for histopathological analysis. Results were peer reviewed by a veterinary pathologist at NIOSH (Morgantown, WV). Images were captured using an Olympus BX53 microscope equipped with a DP73 camera.

### Enhanced-darkfield light microscopy imaging of nanoparticles

CoO and La_2_O_3_ nanoparticles in sections from exposed lungs and tracheobronchial lymph nodes were assessed using an enhanced-darkfield optical system as described previously [14]. Although these nanoparticles have dimensions less than the wavelength of light, they have closely packed atoms, and thus typically have a refractive index significantly different from that of biologic tissues and/or mounting medium. These characteristics produce significantly greater scattering of light by nanoparticles, which then appear as bright particles compared to the surrounding tissues. As described previously, this method of imaging can be used to scan lung sections at relatively low magnification to identify nanoparticles that would not be detected by other means [13–16].

The optical system for detection of nanoparticles consisted of high signal-to-noise, darkfield-based illumination optics adapted to an Olympus BX-41 microscope (CytoViva, Auburn, AL). Sections for darkfield examination were specifically cut from paraffin blocks and collected on ultrasonically cleaned, laser cut slides (Schott North America Inc, Elmsford, NY) to avoid nanoparticle contamination from the ground edges of traditional slides. After staining with Sirius red-hematoxylin, sections were coverslipped with Permount. After alignment of the substage oil immersion optics with a 10× objective, sections were examined with 60× air or 100× oil immersion objectives. Enhanced darkfield images were taken with a 2048 × 2048 pixel digital camera (Dage-MTI Excel digital camera XLMCT, Michigan City, IN).

### Statistics

Statistical comparisons for CoO and La_2_O_3_ nanoparticle exposed groups, separately, across three concentrations including air controls were performed separately for each of three post exposure times using analysis of variance (ANOVA). Since variance estimates were different across treatment groups, the ANOVA models were estimated using an unequal variance method available from SAS PROC MIXED [17]. Similarly, comparisons across exposure times for each concentration were performed using unequal variance ANOVA. All statistical tests were two tailed with the significance level set to 0.05.

## Results

### Aerosol particle characterization

**Fig. 1:**
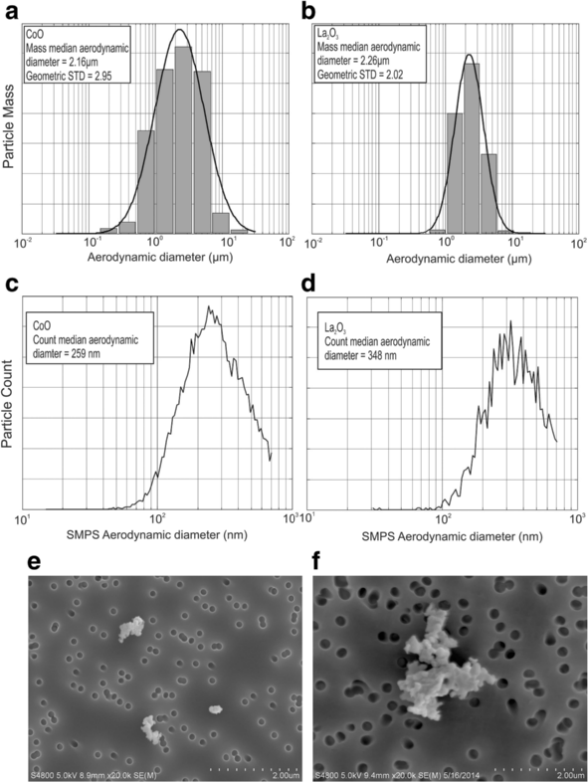
Aerosol particle characterization. Mass median aerodynamic diameter of **a** CoO and **b** La_2_O_3_ nanoparticles measured by a cascade impactor (MOUDI). Count median aerodynamic diameter of **c** CoO and **d** La_2_O_3_ nanoparticles measured by a scanning mobility particle sizer. FESEM of aerosolized **e** CoO and **f** La_2_O_3_ nanoparticles from the whole-body inhalation chamber

Exposure chamber aerosol samples were collected by drawing particles from the chamber through several aerosol instruments. The mass median aerodynamic diameters of the CoO and La_2_O_3_ nanoparticles were determined by a cascade impactor (MOUDI Models 110-R, MSP Corp, Shoreview, MN), which fractionates the particles into 10 size ranges from 56 nm to 18 μm. Results are depicted in Fig. 1a and b. The mass median aerodynamic diameter of CoO nanoparticles was determined to be 2.16 μm with a geometric standard deviation of 2.95. The mass median aerodynamic diameter of La_2_O_3_ nanoparticles was determined to be 2.26 μm with a geometric standard deviation of 2.02. The count median aerodynamic diameters of the CoO and La_2_O_3_ nanoparticles were measured using a Scanning Mobility Particle Sizer (SMPS model, TSI Inc, Shoreview, MN). These data are depicted in Fig. 1c and d. The median mobility equivalent diameter was 259 nm for CoO nanoparticles and 348 nm for La_2_O_3_ nanoparticles, with geometric standard deviation of 1.65 nm and 1.53 nm for CoO and La_2_O_3_ nanoparticles, respectively. Aerosol samples were collected on 25 mm polycarbonate filters with 0.2 μm diameter pores (EMD-Millipore, Billerica, MA) at a rate of 1 L/min. Particle imaging was performed using these filter samples of CoO and La_2_O_3_ nanoparticles with a field emission scanning electron microscope (FESEM; JEOL 6400, JEOL Inc) (Fig. 1e and f, respectively).

### Lung burden and clearance of CoO and La_2_O_3_ nanoparticles

**Fig. 2:**
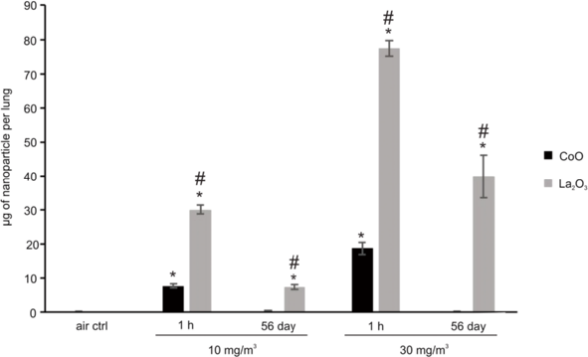
Whole lung burden and clearance of CoO and La_2_O_3_ nanoparticles. Lungs and associated lymph nodes from mice were analyzed by INAA for initial whole lung burden (1 h post exposure on day 4) and lung clearance (56 days post exposure) for CoO and La_2_O_3_ nanoparticles at 10 and 30 mg/m^3^. Values are presented as μg of nanoparticle per weight of lung. *n* = 5 male mice per group. *p < 0.05 represents significance between air control and CoO or La_2_O_3_ nanoparticle-exposed mice. # *p* < 0.05 represents significance between CoO and La_2_O_3_ nanoparticle-exposed mice

Lungs, consisting of the lower trachea, lung lobes and lymph nodes, were analyzed to determine the whole lung burden of CoO and La_2_O_3_ nanoparticles by INAA at 1 h after completion of the inhalation exposure. As seen in Fig. 2, mice exposed to CoO and La_2_O_3_ nanoparticles at 10 mg/m^3^ had an initial whole lung burden of 7.7 μg/lung and 30.1 μg/lung, respectively. At 30 mg/m^3^, CoO nanoparticle-exposed mice had a whole lung burden at 1 h post exposure of 18.7 μg/lung, while La_2_O_3_ nanoparticle-exposed mice had a whole lung burden of 77.5 μg/lung 1 h after exposure. To determine nanoparticle clearance, whole lungs from mice exposed to the low or high particle doses were examined at 56 days post exposure. CoO nanoparticles were rapidly cleared, to the extent that <1 % of the nanoparticles remained in the lung plus lymph nodes at both exposure concentrations. However, for La_2_O_3_ nanoparticles, 22 and 50 % of the particles remained in the lung and lymph nodes 56 days after low and high dose exposures to La_2_O_3_ nanoparticles, respectively. These data indicate considerable differences in the rate of CoO and La_2_O_3_ nanoparticle clearance.

**Fig. 3:**
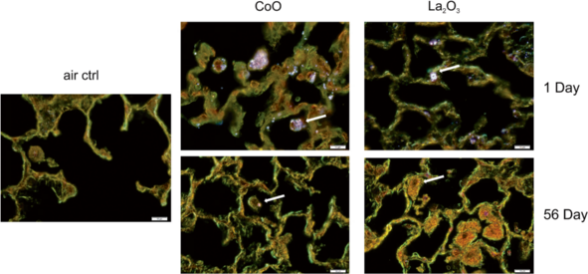
Enhanced-darkfield images-lung deposition. Representative images of lungs from mice 1 and 56 days after exposure to 30 mg/m^3^ illustrate the lung burden and clearance of nanoparticles from the lung. *n* = 6 male mice per group. Nanoparticles are identified by white arrows

**Fig. 4:**
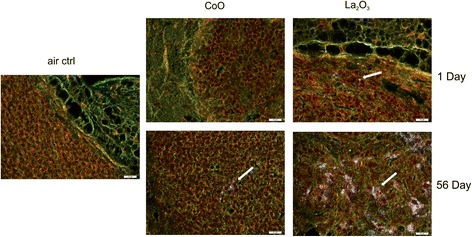
Enhanced-darkfield images- tracheobronchial lymph nodes. Representative images of tracheobronchial lymph nodes from mice 1 and 56 days post exposure to 30 mg/m^3^ illustrate the initial lung burden and accumulation of nanoparticles in the tracheobronchial lymph nodes. *n* = 6 male mice per group. Nanoparticles are identified by white arrows

**Fig. 5:**
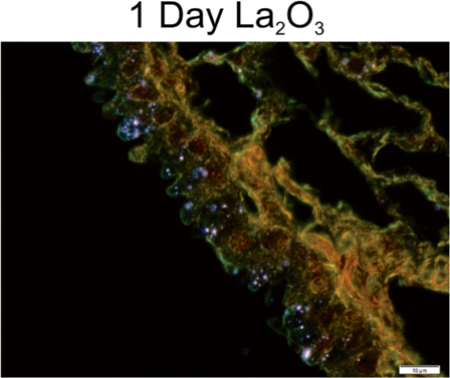
Enhanced-darkfield images-airway epithelium. Representative images of the accumulation of La_2_O_3_ nanoparticles in the airway epithelium from mice at 1 day post exposure to 30 mg/m^3^. *n* = 6 male mice per group

To further investigate the lung burden and clearance of CoO and La_2_O_3_ nanoparticles, enhanced-darkfield light microscopy was used to analyze the lungs and tracheobronchial lymph nodes for nanoparticle deposition and accumulation in lymph nodes in mice exposed at 30 mg/m^3^. CoO and La_2_O_3_ nanoparticles were present in the lung tissue at 1 day post exposure (Fig. 3). An equal amount of La_2_O_3_ nanoparticles was found in the alveolar macrophages when compared to the CoO nanoparticles; however, more La_2_O_3_ nanoparticles were seen in the interstitium at 1 day post exposure, reflecting a higher lung burden 1 h post exposure and more translocation of La_2_O_3_ nanoparticles. These data correlated with the whole lung burden results measured by INAA, indicating greater clearance of CoO nanoparticle than the La_2_O_3_ nanoparticles. Therefore, the tracheobronchial lymph nodes were analyzed (Fig. 4). Results showed that the lymph nodes had no CoO nanoparticles, but showed some scattered La_2_O_3_ nanoparticles at 1 day post exposure. Furthermore, the lymph nodes of mice exposed to La_2_O_3_ nanoparticles at 56 days post exposure showed abundant nanoparticles, supporting migrating of La particles from the interstitium to the lymphatics. In contrast, the lymph nodes of only 2 of the 5 animals examined showed a few scattered CoO nanoparticles at 56 days post exposure. In the conducting airways, it was observed that the CoO nanoparticles were cleared very rapidly (data not shown), while the La_2_O_3_ nanoparticles accumulated in the airway epithelium at 1 day post exposure for the high-dose exposure group (Fig. 5). Therefore, CoO nanoparticles depositing on the conducting airways appear to be cleared rapidly during the 4 day inhalation exposure by the mucocillary escalator, while La_2_O_3_ nanoparticles clearance from the conducting airways are inhibited by rapid uptake by airway epithelial cells. These enhanced darkfield results support the INAA data showing that the whole lung burden of CoO nanoparticles 1 h post exposure is lower than that of La_2_O_3_ nanoparticles. Additionally, La_2_O_3_ nanoparticles appear to translocate to tracheobronchial lymph nodes, resulting in relatively high whole lung burdens at 56 days post exposure.

### Transformation of La_2_O_3_ nanoparticles in phagolysosomal simulated fluid (PSF)

**Fig. 6:**
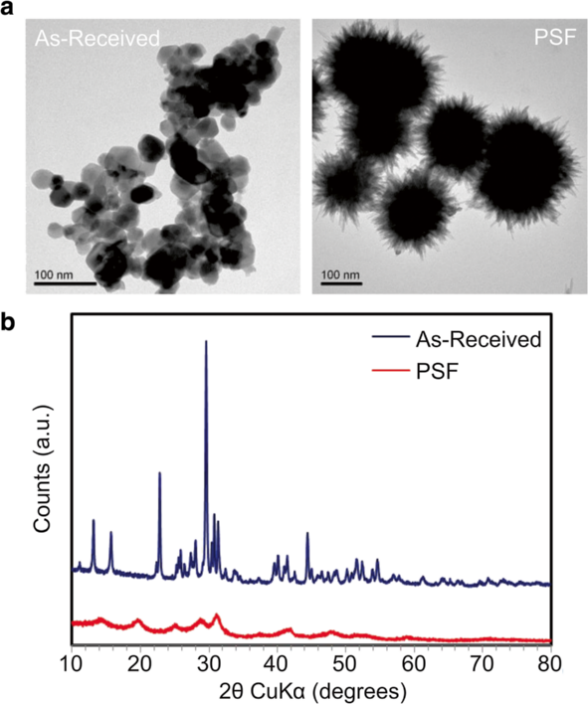
Transformation of La_2_O_3_ nanoparticles in phagolysosomal simulated fluid (PSF). **a** TEM images and **b** XRD spectra of La_2_O_3_ nanoparticles after PSF exposure. La_2_O_3_ nanoparticles were suspended in H_2_O or PSF at 50 μg/mL and incubated at 37 °C for 24 h. After washing in H_2_O, the morphological and crystal structure changes in La_2_O_3_ nanoparticles were examined by TEM and XRD, respectively

As we reported previously, exposure of differentiated THP-1 macrophages to La_2_O_3_ nanoparticles (50 μg/ml) resulted in engulfment and incorporation of these nanoparticles into phagolysosomal vesicles after 6 h in culture [6]. Suspension of La_2_O_3_ nanoparticles in an acidic PSF for 24 h resulted in a change in nanoparticle morphology from spheres to a sea-urchin-like shape (Fig. 6a). This morphological transformation is associated with a chemical modification of the nanoparticle from La_2_O_3_ to LaPO_4_ as determined by XRD (Fig. 6b). In contrast, CoO nanoparticles do not undergo phosphate complexation and do not undergo transformation. We have previously demonstrated that La_2_O_3_ nanoparticles undergo similar transformation in the lung [6] and proposed that the accompanying insolubility of the LaPO_4_ may be responsible for slow clearance of these particles, allowing their translocation across the epithelial layer to the interstitium and from there to the regional lymph nodes.

### Pulmonary damage and inflammation

**Fig. 7:**
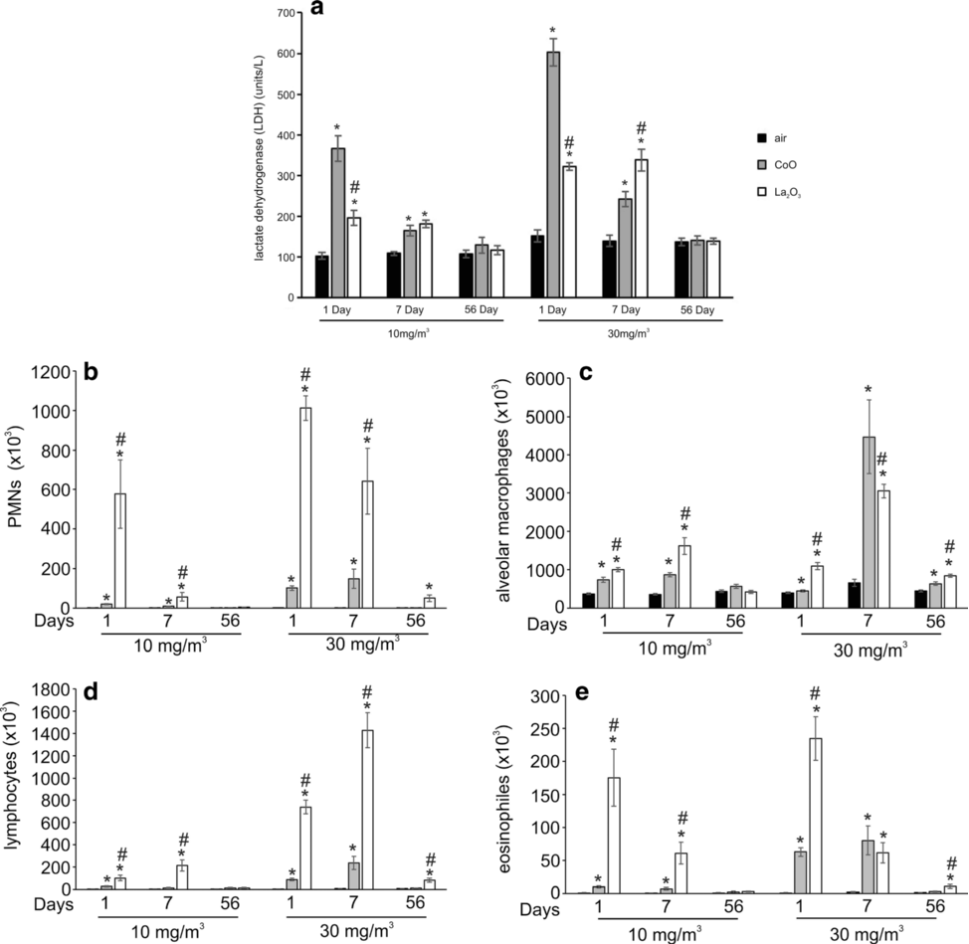
Pulmonary damage and inflammation. BALF was analyzed for **a** lactate dehydrogenase activity and cell differentials from mice exposed to air control, CoO or La_2_O_3_ nanoparticles at 1, 7 and 56 day post exposure. **b** PMNs, **c** alveolar macrophages, **d** lymphocytes, and **e** eosinophils were counted from each BAL cytospin. Values are mean ± standard error. *n* = 4 mice per group. * *p* < 0.05 represents significance between air control and CoO or La_2_O_3_ nanoparticle-exposed mice. # *p* < 0.05 represents significance between CoO and La_2_O_3_ nanoparticle-exposed mice

LDH activity in BALF was used as a measure of nanoparticle-induced pulmonary damage at days 1, 7 and 56 post exposure. Figure 7a shows that CoO and La_2_O_3_ nanoparticles induce significant LDH release at 1 and 7 days post-exposure compared to the air-only control, but returned to basal levels by 56 days post exposure for both low- and high-dose exposures. When comparing CoO and La_2_O_3_, CoO nanoparticles resulted in significantly higher LDH levels 1 day after the 10 mg/m^3^ exposure than La_2_O_3_ nanoparticles. However, there were no further differences between these nanoparticles for low-dose exposures at all other post-exposure times. When comparing the impact of high-dose exposures, CoO nanoparticles significantly increased LDH levels at 1 day post exposure compared to La_2_O_3_ nanoparticles, but this position was reversed at 7 days post exposure, when La_2_O_3_ nanoparticles induced greater LDH release compared to CoO nanoparticles. There were no significant differences between nanoparticle-exposed groups and the air control at 56 days post inhalation for both low and high exposure levels.

Pulmonary inflammation was determined as polymorphonuclear leukocytes (PMNs) calculated from total BAL cell counts and cell differentials of BAL samples from exposed mice. As seen in Fig. 7b, PMNs were significantly elevated at 1 and 7 days post exposure for low-dose exposure to both particle types compared to the air-only control. While the PMNs cell count was higher in La_2_O_3_ nanoparticles compared to CoO nanoparticle-exposed animals at 1 and 7 days post exposure, the counts returned to background levels at 56 days post-exposure. The same pattern was seen for high-dose exposures on days 1 and 7 days post exposure. However, 56 days after the high-dose La_2_O_3_ nanoparticles exposure, a significant increase in the BALF PMNs cell counts persisted when compared to the air-only control. This was not the case for high-dose exposure to CoO nanoparticles. Figure 7c indicates that there were increased numbers of alveolar macrophages during low-dose exposure to both particle types at day 1 and 7 post-exposure, as well as, after 1, 7 and 56 days following exposure to 30 mg/m^3^ to both particle types. When comparing the number of alveolar macrophages in CoO and La_2_O_3_ nanoparticle-exposed BAL samples, La_2_O_3_ nanoparticles had significantly higher counts at 1 and 7 day post exposure at the low exposure and at 1 and 56 day after the higher exposure level. Figure 7d demonstrates that low-dose CoO nanoparticles exposure induced higher lymphocyte cell counts at 1 day post exposure, as well as, on days 1 and 7 following high-dose exposure. La_2_O_3_ nanoparticles induced increased lymphocyte cell counts on days 1 and 7 after low-dose exposure and on days 1, 7 and 56 after high-dose exposure. CoO and La_2_O_3_ nanoparticles also induced elevated eosinophils levels on days 1 and 7 following both low-and high-dose exposures. Eosinophils levels remained elevated 56 days after high-dose La_2_O_3_ nanoparticles exposure (Fig. 7e). When contrasting the induction of eosinophils between CoO and La_2_O_3_ nanoparticles, it was determined that La_2_O_3_ nanoparticles induced higher counts at days 1 and 7 in response to low-dose exposure, as well as 1 and 56 days after high-dose exposure. In summary, these data indicate that while both CoO and La_2_O_3_ nanoparticles induced acute pulmonary inflammation, only La_2_O_3_ nanoparticles induced a chronic inflammatory response with an increased recruitment of a spectrum of inflammatory cells when compared to both air control and CoO nanoparticle-exposed mice.

### CoO and La_2_O_3_ nanoparticle-induced cytokine production

**Fig. 8:**
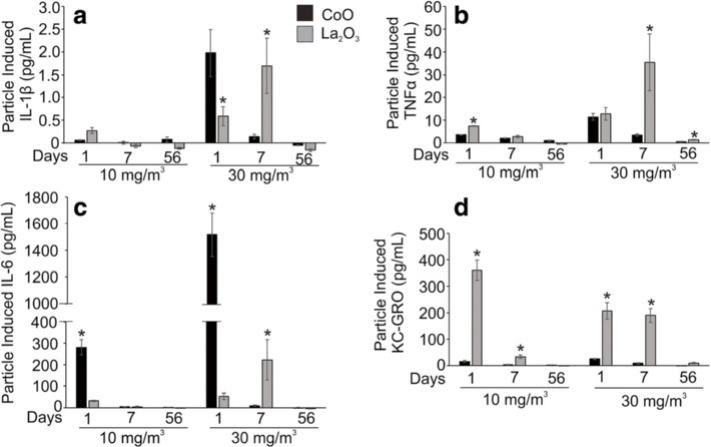
CoO and La_2_O_3_ nanoparticle-induced pro-inflammatory response in BALF. **a** IL-1β, **b** TNFα, **c** IL-6 and **d** KC/GRO were all analyzed in the BALF of exposed mice. The measurement of cytokines was performed on BALF at both doses and all time points. Values are mean ± standard error. *n* = 4 mice per group. * *p* < 0.05 represents significance between air control and CoO or La_2_O_3_ nanoparticle-exposed mice. # *p* < 0.05 represents significance between CoO and La_2_O_3_ nanoparticle exposed mice

**Fig. 9:**
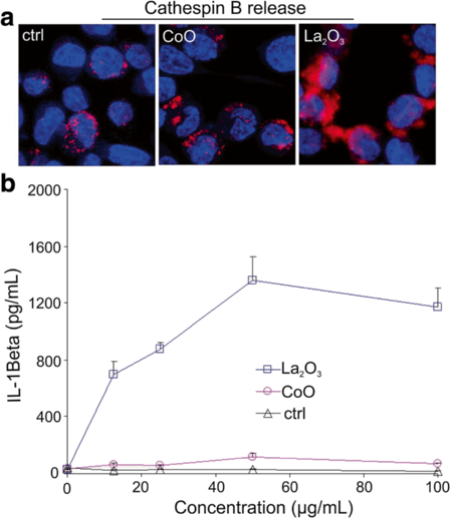
Lysosomal damage, cathepsin B release and IL-1β production in THP-1 cells. THP-1 cells were incubated with 50 μg/mL CoO or La_2_O_3_ nanoparticles for 6 h. Then cells were stained with Magic Red-labeled cathepsin B substrate and visualized by confocal microscopy (**a**). IL-1β production was assessed by ELISA after THP-1 cells were treated with 0–100 μg/mL CoO or La_2_O_3_ nanoparticles for 24 h. (**b**)

Ten different cytokines were examined in the BALF of mice exposed to low and high doses of CoO and La_2_O_3_ nanoparticles, and sacrificed at 1, 7 and 56 days post exposure. Four cytokines showed significant differences from the air-only control. This includes increased release of IL-1β in the BALF on days 1 and 7 after high-dose exposure to both nanoparticle types (Fig. 8a). IL-1β levels were higher 1 day after CoO nanoparticles exposure compared to La_2_O_3_ nanoparticles. However, the opposite was seen after 7 days, with La_2_O_3_ nanoparticles inducing the greater response. After 56 days, there were no increases in IL-1β levels for either nanoparticle type. In order to elucidate the possible explanation for the differences in sustained in vivo IL-1β production in response to the different particle types, we performed an in vitro study with differentiated THP-1 macrophages. We have previously demonstrated that upon differentiation of this myeloid cell line, THP-1 cells attain macrophage-like properties. Therefore, differentiated THP-1 macrophages have commonly been used to assess responses of pulmonary macrophages to a number of engineered nanoparticles. Since both CoO and La_2_O_3_ nanoparticles have been shown by our lab to be engulfed by differentiatedTHP-1 macrophages and become associated with phagolysosomes [6], we assessed whether there is a difference in their ability to induce lysosomal damage by using a Magic Red-labeled substrate for the lysosomal enzyme, cathepsin B. As shown in Fig. 9a, both non-treated, as well as, CoO nanoparticle-treated cells exhibit a punctate distribution of Magic Red, which is confined to intact lysosomes. However, in the case of La_2_O_3_ nanoparticle-treated cells, the dye could be seen to spread diffusely to the cytosol, indicating lysosomal damage. Lysosomal damage, as well as the release of cathepsin B are known stimuli that lead to the assembly of the NLRP3 inflammasome, which is responsible for cleavage of pre-IL-1β to IL-1β. Accordingly, we also found that in vitro exposure to La_2_O_3_ nanoparticles induced a significant increase in IL-1β production compared to CoO nanoparticles, which induced little effect (Fig. 9b).

TNFα (Fig. 8b) was significantly elevated at 1 and 7 days post exposure for both nanoparticle types at low and high doses, compared to the air-only control. Moreover, the levels remained higher than control for up to 56 days following the high-dose La_2_O_3_ nanoparticles exposure. La_2_O_3_ nanoparticles induced higher TNFα level than CoO nanoparticles 1 day after low-dose exposure and 7 days after high-dose exposure. IL-6 (Fig. 8c) was increased at 1 day after the low-dose exposure and days 1 and 7 following high-dose exposure for both nanoparticle types. CoO nanoparticles induced a bigger response than La_2_O_3_ nanoparticles 1 day after exposure, while La_2_O_3_ nanoparticles induced the greater response 7 days after high-dose exposure. KC/GRO (Fig. 8d) was significantly elevated by CoO nanoparticles at 1 and 7 days after both exposure levels. La_2_O_3_ nanoparticle induced KC/GRO expression at 1 and 7 days after both exposure levels and at 56 days after the high exposure compared to air control. When comparing the particle responses, low-dose La_2_O_3_ nanoparticles induced greater KC/GRO expression than CoO nanoparticles at 1 and 7 days post exposure, as well as at all time points after high-dose exposure. Taken together, these data suggest that the different expression patterns of pro-inflammatory cytokines and chemokines in the BALF of mice exposed to CoO and La_2_O_3_ nanoparticles may lead to different modes of pulmonary damage and inflammation.

### Histopathology

**Fig. 10:**
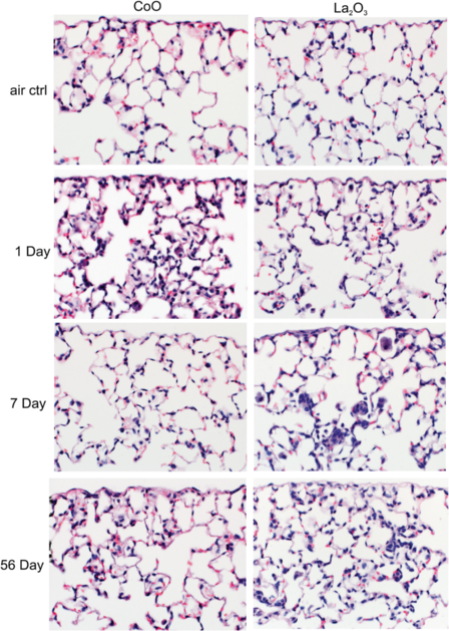
Histopathology. Lungs of mice exposed to 30 mg /m^3^ CoO or La_2_O_3_ nanoparticles or air control were stained with H&E. *n* = 12 mice per group

Mouse lungs were analyzed histologically after animal sacrifices on days 1, 7, and 56 following low and high-dose exposures to both particle types. There were no significant pathological changes observed at low exposure levels (data not shown). However, after high-dose exposure, we observed an increase in the number of alveolar macrophages in response to CoO nanoparticles inhalation at 1 and 7 days post exposure, which returned to air-only control levels by day 56. In contrast, the number of alveolar macrophages in lungs of La_2_O_3_ nanoparticle-exposed mice increased progressively from day 1 to day 7 post exposure and remained elevated for up to 56 days post exposure (Fig. 10).

**Fig. 11:**
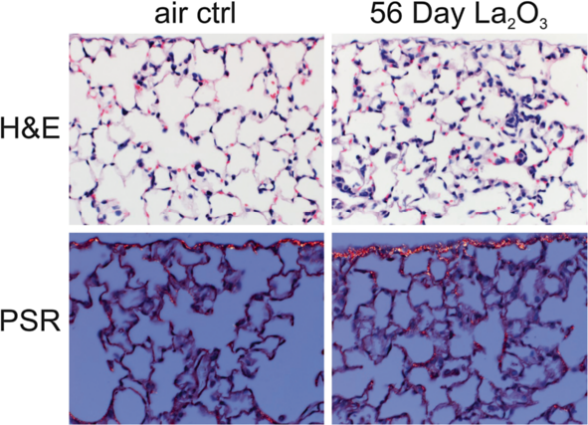
Interstitial fibrosis in lungs of La_2_O_3_ nanoparticle-exposed mice. Higher power magnification and Picrosirius red (PSR) staining demonstrate a small focus of interstitial fibrosis in a La_2_O_3_ nanoparticles exposed animal at 56 days post exposure. *n* = 12 male mice per group

There was no prominent interstitial fibrosis observed in the lungs of animals exposed to CoO nanoparticles 56 days after inhalation exposure. In contrast, minimal fibrosis was occasionally seen in the lungs of animals exposed to La_2_O_3_ nanoparticles 56 days post exposure (Fig. 11). This fibrosis was localized to areas of inflammation, suggesting collagen deposition at sites of macrophage activation and cytokine release. Infrequent observations in the lung tissue of the La_2_O_3_ nanoparticle-exposed animals, included epithelial hyperplasia of bronchioles and alveoli, vasculitis (data not shown), and pleural fibrosis (Fig. 11). There were no observable differences seen in the lungs of CoO nanoparticle-exposed mice at 56 day post exposure compared to air control (data not shown).

## Discussion

The present study determined pulmonary responses to inhalation exposure to CoO and La_2_O_3_ nanoparticles. Exposures were 6 h/day for 4 days to 10 mg/m^3^ or 30 mg/m^3^. Lung burden was measured at 1 h and 56 days post-exposure to evaluate clearance. Bronchoalveolar lavage was conducted to evaluate lung damage (LDH) and inflammation (cell differentials and cytokines) at 1, 7, and 56 days after inhalation exposure. Enhanced darkfield microscopy was conducted at 1 and 56 days post exposure to determine initial deposition and time-dependent translocation of the nanoparticles. Histopathologic analysis determined the degree of pulmonary inflammation and fibrosis at 1, 7, and 56 days after inhalation exposure to these nanoparticles.

Inhalation of CoO nanoparticles resulted in acute pulmonary damage and inflammation with BAL levels of LDH and inflammatory mediators peaking at 1 day post-exposure (Figs. 7 and 8). Likewise histopathologic results indicate an alveolar macrophage inflammatory response, which peaked at 1 day after inhalation of CoO nanoparticles (Fig. 10). In contrast, pulmonary responses to inhalation of La_2_O_3_ nanoparticles were more persistent than after CoO nanoparticles exposure. Indeed, LDH activity of BALF and levels of inflammatory mediators peaked at 7 days post exposure (Figs. 7 and 8), and histopathology indicated that mild histocytosis was found in La_2_O_3_ nanoparticle-exposed lungs at 56 days after inhalation exposure. In addition, La_2_O_3_ nanoparticles exposure resulted in a mild fibrotic response (Fig. 11).

In summary, the present data concerning pulmonary responses 56 days after in vivo exposure suggest that inhalation exposure to La_2_O_3_ nanoparticles was more fibrogenic and caused more persistent inflammation than CoO nanoparticles at equivalent aerosol exposure levels. In comparison, our previous in vitro assays of cytotoxicity and induction of oxidative stress reported that CoO nanoparticles were substantially more potent than La_2_O_3_, supporting predictions from band gap theory [3, 7]. Additionally, our short term (1 day post) oropharyngeal aspiration study in mice reported that CoO nanoparticles caused a greater acute inflammatory response, supporting the conclusion from in vitro studies [3]. It is likely that differences between our previous in vitro and acute (1 day post exposure) in vivo studies and those of the present longer term (56 day post-exposure) 4 day inhalation exposure study are due to differences in the clearance rates for these two nanoparticles. Measurement of lung burdens 1 h after the end of the 4 day inhalation exposure indicate that burdens for CoO nanoparticles were only 25 % of those for La_2_O_3_ nanoparticles at both the low (10 mg/m^3^) and high (30 mg/m^3^) exposure levels, i.e., lung burdens of 7.7 and 18.7 μg vs. 30.1 and 77.5 μg for CoO and La_2_O_3_ nanoparticles, respectively. These differences in lung burdens at the start of the post exposure observation period would not have been predicted by the lung deposition vs particle aerodynamic diameter data reported by Raabe et al. [18] for mice, since aerosol levels and mass median aerodynamic diameters (MMADs) for CoO and La_2_O_3_ nanoparticles were nearly identical for these inhalation exposures (Fig. 1). Of note, estimations from the Raabe et al. data predicts that as high as 60 % of lung deposition of particles with MMADs of 2 μm would be in the tracheobronchiolar region of the mouse lung, with the remaining 40 % depositing in the alveolar region of the lung [18]. Enhanced darkfield microscopy at 1 day post exposure indicates that CoO nanoparticles were rapidly cleared from the conducting airways during the 4 day inhalation period by the mucocillary escalator, which exhibits a half time for clearance of a few hours. However, enhanced darkfield microscopy indicates that 1 day after inhalation exposure the La nanoparticles had translocated from the surface of the conducting airways into bronchial epithelial cells (Fig. 5c), thus, escaping mucocillary clearance. This emphasizes that calculated lung deposition does not always reflect actual particle lung burdens, and that measurement of lung burden is essential to correctly interpret pulmonary response. Our previous in vitro [7] and 1 day post-aspiration in vivo [3] studies would not be able to discern this clearance-dependent difference in initial lung burden of CoO and La_2_O_3_ nanoparticles; thus, they would fail to predict the greater sub-chronic inflammatory and fibrogenic potential of La_2_O_3_ vs. CoO nanoparticles found in the present study.

As discussed above, lung burden 1 h after the 4 day exposure was greater for La than Co metal oxide nanoparticles, reflecting predominant rapid clearance of CoO nanoparticles by the mucocillary escalator. Concerning the slow phase of clearance, whole lung burden for La nanoparticles was also greater than that for CoO nanoparticles at 56 days post-exposure, where persistent whole lung burden was 6.6 μg and 38.8 μg for the low and high level of La_2_O_3_ nanoparticles, respectively, compared to < 1 % of the 1 h post exposure lung burden for CoO nanoparticles. There are two different slow pulmonary clearance mechanisms: 1) uptake of the nanoparticles deposited in the respiratory zone by alveolar macrophages and slow migration of these particle-laden cells to the mucocillary escalator, or 2) translocation of the nanoparticles across epithelial cells into the interstitium and slow clearance through the lymphatic system [19]. Upon analyzing the enhanced darkfield images, the CoO nanoparticles deposited in the respiratory zone were seen mainly in alveolar macrophages at 1 day post-exposure, suggesting slow clearance via migration to the mucocillary escalator (Fig. 3). However, a large amount of the La nanoparticles were seen in epithelial cells and the interstitium of the airways at 1 day post exposure (Figs. 3 and 5) and in the lymphatic system by 56 days post exposure (Fig. 4). In contrast, very few CoO nanoparticles were detected in the tracheobronchial lymph nodes of exposed mice at 56 days post inhalation. The difference in pulmonary fate and clearance mechanism for Co and La nanoparticles would result in the observed differences in pulmonary responses reported in the present study.

Mechanistic non-animal studies do shed light upon the results of the present mouse inhalation study. For example, we have reported that La_2_O_3_ nanoparticles were present within phagolysosomes of differentiated THP-1 macrophages after a 6 h exposure in culture [6]. Additionally, we showed that La_2_O_3_ nano-spheres transform into sea urchin-shaped LaPO_4_ particles after a 24 h incubation in an acidic PSF, and such sea urchin-shaped particles were found in the differentiated THP-1 macrophages 12 h after in vitro exposure to La_2_O_3_ nanoparticles. Associated with this transformation of La nanoparticles was rupture of lysosomal membranes, demonstrated by the cytosolic release of cathepsin B into the cytoplasm of differentiated THP-1 macrophages 12 h after in vitro exposure to La_2_O_3_ nanoparticles [6]. Our lab has also shown that rupture of lysosomes in alveolar macrophages was observed 40 h after exposure of mice by aspiration of La_2_O_3_ nanoparticles [6]. Therefore, it is feasible that in the present inhalation study that La_2_O_3_ nanoparticles were transformed upon engulfment by alveolar macrophages, ruptured phagolysosomes, escaped these phagocytes, and entered bronchial epithelial cells, thus, avoiding clearance by the mucocillary escalator. CoO nanoparticles, in contrast to La_2_O_3_, did not undergo such transformation, since they did not lyse lysosomes (Fig. 9). Therefore, CoO nanoparticles would remain in macrophages and be cleared.

Mechanistic in vitro studies also predict the fibrotic response to La_2_O_3_ nanoparticles exposure observed in the present inhalation study. Indeed, Fig. 9b shows that in vitro exposure of THP-1 macrophages to La_2_O_3_ nanoparticles for 6 h activated the NLRP3 inflammasome as indicated by a substantial, dose-dependent release of IL-1β with La_2_O_3_ nanoparticles, while CoO nanoparticles failed to induce IL-1β production. Activation of the NLRP3 inflammasome has been linked with pulmonary fibrotic diseases, such as silicosis and asbestosis [20, 21]. In addition, IL-1β has been shown to activate fibroblasts and cause pulmonary fibrotic responses [22–24]. The present study indicates that inhalation of La_2_O_3_ nanoparticles caused a 9 fold greater Il-1β production 7 days after inhalation than the non-fibrotic CoO nanoparticles inhalation (Fig. 8a).

Of interest, our longer term (21 day post exposure) aspiration study in mice did observe NLRP3 inflammasome activation (2.4 fold increase in BALF levels of IL-1β) and pulmonary fibrosis in response to La_2_O_3_ nanoparticles [6]. This indicates that when lung burdens are similar, results of bolus particle exposure studies can be predictive of more costly and technically more complex inhalation studies. Therefore, there is an important role for pulmonary bolus exposure studies (oropharyngeal aspiration and intratracheal instillation) in a tiered approach to hazard ranking and assessment for nanoparticles.

In addition to IL-1β, other mediators have been associated with fibrogenesis. TNF-α has been demonstrated to play a key role in activating molecular pathways leading to pulmonary fibrosis [25–27]. In the present study, we found that La_2_O_3_ nanoparticles inhalation at 30 mg/m^3^ caused a 6 fold greater production of TNF-α than CoO nanoparticles at 7 days post exposure (Fig. 8b). Previously, we reported that aspiration of La_2_O_3_ nanoparticles induced a 2 fold increase in BALF levels of TGF-β1 and PDGF, mediators associated with pulmonary fibrogenesis, 21 days post exposure in mice [6]. This was associated with a 67 % increase in lung collagen.

In the present study, the lung burden of La_2_O_3_ nanoparticles 1 h after a 4 day inhalation of 30 mg/m^3^ was 77.5 μg/whole lung. In our previous oropharyngeal aspiration study, lung burden was 50 μg/lung [6]. It should be noted that such lung burdens are lower than those necessary to cause volumetric overload and impairment of clearance [28]. Indeed, alveolar macrophages were not overloaded with particles (Fig. 3), and inhalation of an equal aerosol exposure dose of CoO nanoparticles did not result in delayed clearance.

An issue is the relevance of these pulmonary doses of La_2_O_3_ nanoparticles to expected worker exposure. Unfortunately, no worker exposure data for nano-La_2_O_3_ are available. To date, the only Recommended Exposure Limit (REL) for a nano metal oxide is 0.3 mg/m^3^ proposed for nano TiO_2_ [29]. Assuming this level of airborne La_2_O_3_ nanoparticles in a workplace, workers would be expected to achieve a lung burden, normalized to an equivalent burden per lung surface area, similar to the mice in these studies in approximately 1 year. Therefore, our studies are relevant to feasible worker exposure.

In the present study, the physical chemical transformation of La_2_O_3_ nanospheres to LaPO_4_ urchin-shaped particles is proposed to be related to the interstitial accumulation of La and fibrosis. Of interest, it has recently been reported that CeO_2_ transforms into urchin-shaped phosphate Ce nanoparticles in vivo [30]. This may explain why CeO_2_ nanoparticles exhibit low in vitro cytotoxicity, yet induce pulmonary fibrosis in a rat model [31]. In light of these findings, further investigation into the phosphation of certain nano metal oxides and resultant alterations in the bioactivity of these transformed particles is warranted.

We have proposed high throughput in vitro screening as an essential component of a tiered testing approach for nanoparticles [11]. Prudent selection of in vitro assays to identify adverse outcome pathways (AOPs) produces potency and mechanistic data in a rapid and low cost manner. Such data can be used for hazard ranking. In addition, in vitro testing can provide guidance concerning which in vivo responses to evaluate. We do not propose use of in vitro data for quantitative risk assessment, since in vitro assays use doses/cell surface area much higher than dose anticipated in the human lung per alveolar epithelial surfaces area. However, when carefully conducted, in vitro results have been able to predict in vivo response to nanoparticles, such as carbon nanotubes and metal oxides [6, 32]. In the present study, doses/culture cell surface area were much higher than lung burdens normalized to the alveolar surface in the mouse. However, in vitro studies did produce mechanistic information useful to interpret the differences in clearance of fibrotic responses of mice after inhalation of CoO and La_2_O_3_ nanoparticles.

## Conclusion

**Fig. 12:**
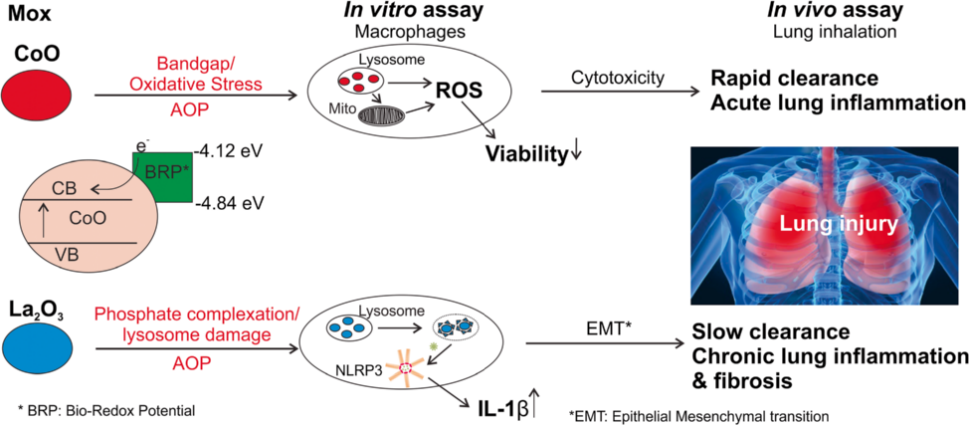
Schematic image showing the toxicity mechanisms of CoO and La_2_O_3_ nanoparticles. The redox potential of CoO nanoparticles falls into the biological redox potential (BRP), which allows electrons to transfer from biological molecules to CoO nanoparticles. This process could induce oxidative stress, leading to mitochondria damage, cell death and acute lung toxicity. In contrast, no electrons were transferred from biological system to La_2_O_3_ nanoparticles. However, La_2_O_3_ nanoparticles could transform into sea urchin LaPO4 in lysosomes, induce lysosomal damage, cathepsin B release, NLRP3 inflammasome activation and IL-1β production, which finally results in chronic lung inflammation and fibrosis

In summary, results from the present study as well as previous studies indicate that a tiered approach is required to predict sub-chronic responses following inhalation of CoO or La_2_O_3_ nanoparticles. Simple in vitro assays for oxidative stress and cell death predict the acute inflammatory response to CoO nanoparticles in agreement with the band gap theory. However, they do not explain the persistent whole lung burden and fibrotic response after inhalation of La_2_O_3_ nanoparticles. More mechanistic in vitro assays associated with biotransformation of La_2_O_3_ nanoparticles to LaPO_4_ and activation of the NLRP3 inflammasome do shed light on the biopersistent and fibrogenic potential of La_2_O_3_ nanoparticles. Lastly, bolus pulmonary exposure studies involving La_2_O_3_ nanoparticles were able to predict the fibrogenic response seen after inhalation exposure, when similar exposure doses and post exposure times are employed. This tiered approach employing relevant adverse outcome pathways for hazard assessment is summarized in Fig. 12.

## Additional file

Additional file 1: Figure S1. Detection of ROS production in nanoparticle suspensions. The oxidative radicals were determined in free cell media (RPMI 1640 supplemented with 10 % serum), 100 μg/mL nanoparticle suspensions by incubating with 25 μg/mL H2DCF solutions. After a 2 h incubation at room temperature, the DCF fluorescence was excited at 490 nm and detected at 520 nm. (TIF 14972 kb)

## Abbreviations

A549: Adenocarcinomic Human Alveolar Base Epithelial Cells
AOPs: Adverse Outcome Pathways
BAL: Bronchoalveolar Lavage
BALF: Bronchoalveolar Fluid
BEAS-2B: Human Epithelial Bronchus Lung Cells
BRP: Biological Redox Potential
Co: Cobalt Monoxide
DNA: Deoxyribonucleic Acid
FESEM: Field Emission Scanning Electron Microscope
H&E: Hematoxylin and Eosin
IFN-γ: Interferon-gamma
IL-10: Interleukin-10
IL-12p70: Interleukin-12
IL-1β: Interleukin-1beta
IL-2: Interleukin-2
IL-4: Interleukin-4
IL-5: Interleukin-5
IL-6: Interleukin-6
INAA: Instrumental Neutron Activation Analysis
KC/GRO: Neutrophil-Activating Protein 3
La_2_O_3_: Lanthanum oxide
LDH: Lactate Dehydrogenase
LPS: Lipopolysaccharide
MMADs: Mass Median Aerodynamic Diameter
MPPD: Multiple-Path Particle Dosimetry
MT3: Metallothionein 3
NIOSH: National Institute for Occupational Safety and Health
NOS2: Nitric Oxide Synthase 2
NRLP3 NLR: Family Pyrin Domain Containing 3
PBS: Phosphate-Buffered Saline
PDGF: Platelet-Derived Growth Factor
PMA: Phorbol 12-Myristate Acetate
PMNs: Polymorphonuclear Leukocyte
PSF: Phagolysosomal Simulated Fluid
PSR: Picrosirius Red
PTG2(COX2): Prostaglandin-Endoperoside Synthase 2
RAW264.7: Mouse Macrophage Cells
REL: Recommended Exposure Limit
ROS: Reactive Oxygen Species
SAEC: Small Airway Epithelial Cells
SOD3: Superoxide Dismutase 3
TEM: Transmission Electron Microscopy
THP-1: Human Monocytic Cells
TiO_2_: Titanium Dioxide
TNFα: Tumor Necrosis Factor-Alpha
XRD: X-Ray Diffraction
